# Aneurysm miRNA Signature Differs, Depending on Disease Localization and Morphology

**DOI:** 10.3390/ijms17010081

**Published:** 2016-01-12

**Authors:** Albert Busch, Martin Busch, Claus-Jürgen Scholz, Richard Kellersmann, Christoph Otto, Ekaterina Chernogubova, Lars Maegdefessel, Alma Zernecke, Udo Lorenz

**Affiliations:** 1 Department for General, Visceral, Vascular & Paediatric Surgery, University Hospital of Würzburg, Würzburg 97080, Germany; kellersman_r@ukw.de (R.K.); otto_c@ukw.de (C.O.); Lorenz_u1@ukw.de (U.L.); 2 Rudolf Virchow-Center, University of Würzburg, Würzburg 97080, Germany; Martin.Busch@med.uni-heidelberg.de; 3 IZKF Laboratory for Microarray Applications, University Hospital Würzburg, Würzburg 97080, Germany; Scholz_C@ukw.de; 4 Department of Medicine, Center for Molecular Medicine (L8:03), Karolinska Institute, Stockholm 12065, Sweden; ekaterina.chernogubova@ki.se (E.C.); lars.maegdefessel@ki.se (L.M.); 5 Institute of Experimental Biomedicine, University Hospital Würzburg, Würzburg 97080, Germany; Zernecke_a@ukw.de

**Keywords:** AAA, popliteal aneurysm, miRNA expression, pathway analysis, histologic diversity

## Abstract

Limited comprehension of aneurysm pathology has led to inconclusive results from clinical trials. miRNAs are key regulators of post-translational gene modification and are useful tools in elucidating key features of aneurysm pathogenesis in distinct entities of abdominal and popliteal aneurysms. Here, surgically harvested specimens from 19 abdominal aortic aneurysm (AAA) and 8 popliteal artery aneurysm (PAA) patients were analyzed for miRNA expression and histologically classified regarding extracellular matrix (ECM) remodeling and inflammation. DIANA-based computational target prediction and pathway enrichment analysis verified our results, as well as previous ones. miRNA-362, -19b-1, -194, -769, -21 and -550 were significantly down-regulated in AAA samples depending on degree of inflammation. Similar or inverse regulation was found for miR-769, 19b-1 and miR-550, -21, whereas miR-194 and -362 were unaltered in PAA. *In situ* hybridization verified higher expression of miR-550 and -21 in PAA compared to AAA and computational analysis for target genes and pathway enrichment affirmed signal transduction, cell-cell-interaction and cell degradation pathways, in line with previous results. Despite the vague role of miRNAs for potential diagnostic and treatment purposes, the number of candidates from tissue signature studies is increasing. Tissue morphology influences subsequent research, yet comparison of distinct entities of aneurysm disease can unravel core pathways.

## 1. Introduction

Abdominal aortic aneurysm (AAA) is a leading cause of cardiovascular death if not diagnosed early and treated surgically by open or endovascular repair [1]. Procedure-related complications create an operation threshold of 50–55 mm [2]. Smaller aneurysms, which are not operated on, have a heterogeneous growth rate and may rupture eventually. Unfortunately, at present neither the growth nor the risk of rupture can be influenced by preventive medical treatment [3,4].

Current understanding of AAA pathogenesis conceives of a multifactorial process, and research has primarily been focused on inflammation and extracellular matrix (ECM) remodeling, evident in human specimens with advanced disease [5,6]. Only recently have the role of alternative mechanisms such as angiogenesis, luminal thrombus pathogenicity and the implications of vascular smooth muscle cell (VSMC) phenotype switching been studied [7,8]. Importantly, aneurysm disease is not limited to the aorta and can also appear universally in the arterial system, including elastic and muscular vessels, such as the popliteal artery (PAA). Genetic profiling of human AAA samples and the discovery of distinct gene sets has broadened the diversity of possible targets to better understand aneurysm pathology. However, except for syndromal disease, such as Marfan syndrome, no single locus has consistently been identified in different AAA cohorts, further supporting the idea that AAA is a multifactorial disease [9,10]. Therefore, a more synergistic approach is necessary to understand aneurysm disease, especially as research is further complicated by its heterogeneous morphology, even within one aneurysm entity [11].

MicroRNAs (miR) are evolutionary conserved, 16–22 nucleotide-long, single-stranded RNA molecules that repress mRNA at the post-transcriptional level. Their contributory role is currently being evaluated in human and murine AAA pathogenesis [12,13]. The polyvalent targeting of miRs allow for a superordinate role in multifactorial diseases [14]. Expression profiles from human thoracic and AAA have revealed multiple candidates, *i.e.*, miR-21, -24, -29, -133, -146, -205, -222 and -712, some of which could even be shown to attenuate AAA formation *in vivo* [15,16,17,18]. The role of miRs as emerging drugs and biomarkers in various fields has gained much attention. An example is the investigation of miR-21in cardiovascular disease, which is also currently in a clinical trial for kidney fibrosis with pending results. Interestingly, the potential of miRs for alternative target and pathway prediction, especially in the setting of distinct entities of aneurysm disease, has only been sparsely addressed.

Therefore, we present miR expression data from human AAA and PAA *vs.* non-aneurysmal vessels in relation to the respective tissue morphology in order to predict aneurysm disease pathways and address the potential and pitfalls of miRNA research in this field.

## 2. Results and Discussion

### 2.1. Results

#### 2.1.1. Candidate miRs for Abdominal Aortic Aneurysm (AAA) Formation

To address previous limitations of studies in human AAA samples, we compared miR expression in aneurysmatic *vs.* non-aneurysmatic abdominal aortae from the same individual in a small sample size of four patients. Corresponding whole tissue specimens were carefully selected on the surgical (macroscopic) and the histological (microscopic) level.

Expression analysis was performed using probes for 758 human miRs, and our analysis revealed 14 significantly differentially expressed candidates, though with great variance (data not shown). The five most differentially regulated miRs, including miR-21 as the most concisely studied miR in cardiovascular disease), were selected for further evaluation in a larger cohort. We also included tissue specimens from PAA patients in order to broaden the view on aneurysm disease.

#### 2.1.2. Down-Regulation of Certain miRNAs Depends on Inflammatory Activity

A total of 19 AAA and 11 atherosclerotic non-aneurysmatic control aortic samples, including the initial specimens, were investigated via qRT-PCR.

**Figure 1:**
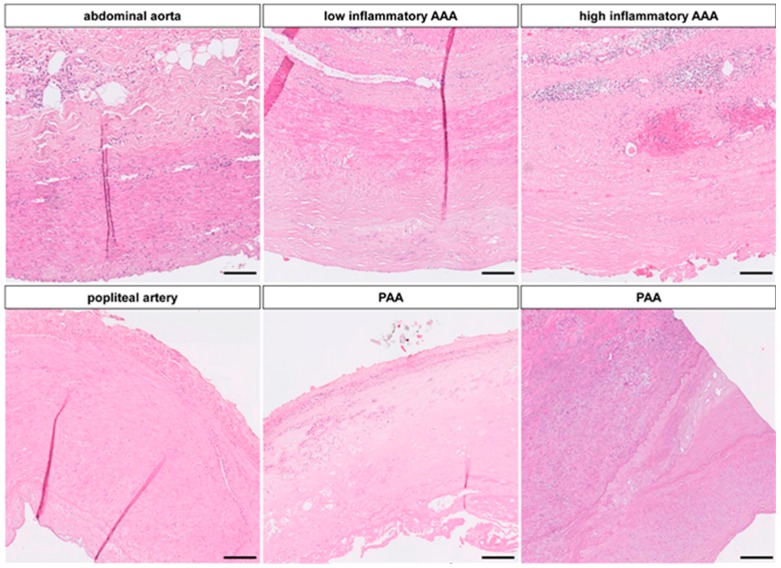
Histologic Scope of Aneurysm Disease: Hematoxylin/Eosin (HE) staining shows the different vessel architecture between elastic (aorta) and muscular (popliteal artery) arteries. The abdominal aorta at the infrarenal position consists of approx. 24 layers of elastic fibers, with a thin endothelium and a clear demarcation to the adventitia with microvasculature, few inflammatory cells and adipocytes, whereas further distally, a single inner and outer elastic lamella make up the margin of the muscular media with contractile vascular smooth muscle cells (VSMC). In aneurysm disease, this morphology changes and the intimal/medial extracellular matrix (ECM) is completely altered by calcification, neoangiogenesis, destruction of elastic fibers, fibrosis and inflammatory infiltration. In aortic aneurysms (**upper** panel) these changes vary, especially in terms of inflammation and fibrosis, whereas the popliteal aneurysm (PAA) morphology is more homogenous (**lower** panel). (magnification 10×, scale bar 50 µm).

Pre-PCR histological assessment of all samples showed a wide variety of AAA morphology based on inflammation, ECM remodeling, calcification, angiogenesis and intima/media-thickness (Figure 1). Low and high inflammatory AAA were distinguished according to the Histologic Inflammation Scale of Aneurysm (HISA) by Rijbroek *et al.* [11] HISA 0/1 were considered low inflammatory and HISA 2/3 were considered high inflammatory (Figure S1). Apart from the current study, tissue heterogeneity was validated in a total of 42 AAA and 12 control aortic samples (data not shown). No significant differences in baseline patients’ characteristics were seen (Table S1).

**Figure 2:**
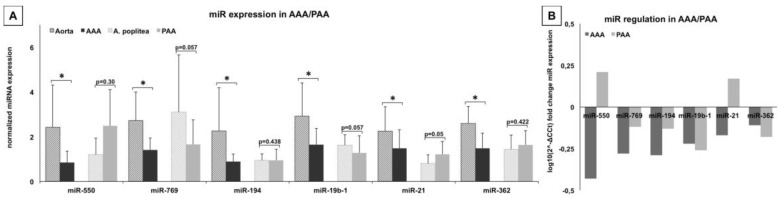

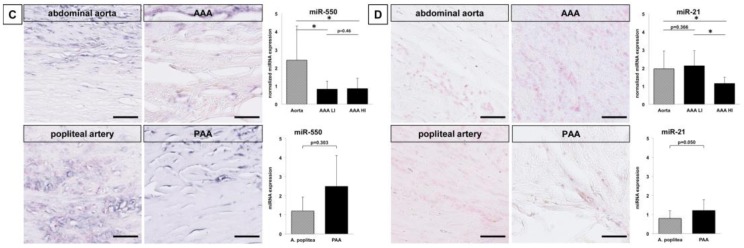
miRNA expression in aneurysm disease: (**A**) miR expression in abdominal aortic aneurysm (AAA), PAA and their respective control tissues normalized to endogenous controls U6 and RNU48 show significant down-regulation for miR-362, -19b-1, -194, -769, -21 and -550 in AAA and down-regulation for miR-19b-1 and -769 in PAA. miR-362 and -194 show no difference and miR-550 and -21 are up regulated in PAA. This is more obvious on the log_10_ fold changes, which highlights the differential regulation of miR-550 and -21 between AAA and PAA; (**B**) Both miRs are located to the VSMCs of the intima/media of the vessel and aneurysm respectively and both show enrichment in PAA in comparison to popliteal artery and AAA/control artoa; (**C**,**D**) While miR-550 is down-regulated in AAA regardless the grade of inflammation, miR-21 shows even up regulation in lower inflammatory specimen compared to high inflammation and control tissue. (*p* = probability; * = *p* < 0.05; magnification 40×; scale bar 100 µm).

Expression analysis revealed significant down-regulation of the six studied miRs, miR-550, -769, -194, -19b-1, -21, -362, in AAA compared to non-aneurysmatic aortae in the cohort of 19 *vs.* 11 specimens (Figure 2). These effects were further analyzed for correlation with grade of inflammation, where regulation of miR-21 and -194 were dependent on inflammatory state, whereas regulation of miR-550, -19b-1, -362, and -769 were independent of inflammatory morphology (Figure 2 and Figure S1).

#### 2.1.3. miR-Signature in Popliteal Artery Aneurysm (PAA) Differs Significantly from AAA

To shed additional light on the implications of these identified miRs in aneurysm disease, we compared miR-expression in paired probes of PAA *vs.* non-aneurysmatic artery.

This rare, yet clinically important, entity showed a more homogenous morphology on the histologic level with complete loss of the muscular artery phenotype (Figure 1). From a total of 15 analyzed samples, 8 were available for miR-expression analysis.

Expression of miR in PAA showed considerably differing trends from AAA tissue, although statistical significance was not achieved (Figure 2). miR-194 and -362 did not differ between aneurysm and control. MiR-550 and miR-21 were up-regulated, miR-19b-1 and -769 were down-regulated in PAA in comparison to non-aneurysmal control tissue.

Since miR-550 and miR-21 were inversely regulated in the two aneurysm entities, these were further analyzed by *in situ* hybridization on aneurysmal and control tissue. Both RNA-molecules showed nuclear localization in VSMCs of the intima/media area and, on a visual level, were verified to be less expressed in AAA compared to PAA (Figure 2).

#### 2.1.4. Signal Transduction, Tissue Remodeling and Cell Interaction Pathways Are Key in Aneurysm Pathogenesis

To relate our results to implications for aneurysm pathogenesis, a miR target prediction using the DIANA miRPath online software was used to identify genes and consecutive pathway enrichment with miR-362, -19b-1, -194, -769, -21 and -550 interactions.

A total of 1859 possible gene targets by at least one of the respective miRs were identified by the algorithm, of which 995 (53%) were for miR-19b-1. Enrichment analysis applying the KEGG database displayed the highest correlation for signal transduction, tissue remodeling and cellular interaction pathways. Inflammatory and cell degrading signaling pathways were fewer and metabolism pathways were of minor importance (Table 1).

A consecutive parallel analysis of previously published miRs from human AAA tissue including miR-29a/b/c, -155, -205, -516a showed similar results highlighting the role of the identified pathways (Table 1) [17,19,20,21]. The most highly ranked pathways in both analysis included “mTOR/TGFß-signaling”, “tight/adherence junction” and “ubiquitin-mediated proteolysis”.

To assess the biological relevance of these *in silico* predictions, we checked protein alteration via immunhistochemistry. CD31 staining for mature endothelial cells confirmed a higher abundance of neoangiogenesis (“VEGF signaling”) in the aneurysmal adventitia, media and neointima and also verified luminal endothelial disruption by loss of CD31 positivity in AAA as well as PAA (“tight/adherence junction”). Additionally TGFß1 expression (“TGF-ß signaling”) showed less protein in the aneurysmal tissue, especially in the medial layer (Figure S1).

**Table 1:** miRNA associated pathways in Aneurysm Disease: KEGG Pathways with predicted interaction enrichments for miR-362, -19b-1, -194, -769, -21, -550 (results from this study) and for miR-29a/b/c, -155, -205, -516a (previously published results). Ln(*p*-value) is a natural logarithmic scale of the *p*-value obtained from a χ-square test comparing expected number of genes with interaction and the actual number composing the pathways (DIANA algorithm). The higher value thus indicates a larger number of genes within the pathway predicted to be regulated by at least one of the respective miRs. Values ranged from 17.64 to 0.01 (3rd column) and 13.13 to 0.04 (4th column). The numbers in brackets indicate the number of gene interactions within the pathway respectively. Note that mTOR- and TGFß-signaling, adherence junction and focal adhesion and ubiquitin-mediated proteolysis receive concordantly high scores in both analyses.

Mode of Interaction	KEGG Pathway	miR Identified in This Study: miR-21, -19b, -194, -362, -769, -550 (in *p*-Value)	miR Identified in Other Studies: miR-29a/b/c, 155, -205, -516a (in *p*-Value)
Signal transduction	MAPK signaling (04010)	22.08 (53)	6.47 (39)
	mTOR signaling (04150)	15.97 (16)	10.92 (14)
	Wnt signaling (04310)	12.91 (31)	4.06 (23)
	ErbB signaling (04012)	12.9 (22)	5.44 (17)
	TGF-ß signaling (04350)	12.29 (22)	7.55 (19)
	JAK-stat signaling (04630)	7.04 (27)	1.69 (2)
	Hedgehog signaling (04340)	6.79 (13)	0.1 (15)
	Calcium signaling (04020)	3.26 (24)	0.47 (17)
	Cytokine cytokine interaction (04060)	0.44 (27)	1.63 (31)
	p53 signaling (04115)	0.05 (7)	1.36 (10)
Cell-Cell interaction	Adherence junction (04520)	4.59 (14)	5.71 (15)
	Tight junction (04530)	3.7 (21)	8.42 (26)
	Gap junction (04540)	3.66 (16)	0.85 (12)
	Cell adhesion molecules (04514)	0.11 (12)	1.19 (16)
Cell-ECM interaction	Focal adhesion (04510)	14.61 (39)	10.72 (36)
	ECM receptor interaction (04512)	3.42 (14)	13.13 (21)
Cell degradation	Ubiquitin mediated proteolysis (04120)	13.46 (29)	4.02 (21)
	Apoptosis (04210)	2.33 (13)	1.57 (12)
	NKcell mediated cytotoxicity (04650)	0.06 (12)	0.08 (11)
Cell growth	Insulin signaling (04910)	9.08 (27)	4.81 (23)
	VEGF signaling (04370)	7.66 (16)	1.21 (10)
	Regulation actin cytoskeleton (04810)	5.39 (32)	2.68 (28)
Inflammation	B-cell receptor (04662)	5.59 (13)	2.13 (10)
	T-cell receptor (04660)	3.24 (15)	6.28 (18)
	Toll like receptor signaling (04620)	2.89 (16)	0.95 (13)
	Coagulation cascade (04610)	0.94 (4)	1.62 (3)
	Leukocyte migration (04670)	0.6 (13)	0.21 (12)

### 2.2. Discussion

Comparing miR expression in degenerative, non-genetic AAA and PAA to verify general features in aneurysm disease is a unique approach presented here for the very first time, and the identified pathways add significantly to further research in the field.

In the past, different approaches have led to a variety of miR signatures in human and rodent AAA being identified, and these are still growing in number [19,22]. Results from successful murine aneurysm models where miRs -21, -24, -29, -205, or -712 abrogated AAA diameter in turn do not match these signatures concisely [15,16,17,18,22]. Furthermore, there is considerable heterogeneity regarding differentially regulated miR candidates between different reports. Human studies are generally limited by a heterogeneous control group of autopsy and/or non-corresponding aortic samples, bearing the risk of RNA decay on the one hand and a different developmental background for tissue-specific miR signatures on the other [21,23]. This is further aggravated by the presence of different histological subtypes of aneurysms, a problem rarely addressed in AAA research (Figure 1 and Figure S1).

Of the miRs identified in our expression profile, only miR-21 has been previously linked to AAA formation (Table 1) [16]. The miR-17-92 cluster, including miR-19b-1, however has been suggested [24,25]. No specific other cluster within 10,000 nucleotides based on miRBase analysis (http://www.mirbase.org/) was represented by any of the identified miRs (data not shown) [26].

After identification of valuable candidates by expression profiling of AAA and control non-aneurysmatic samples from the same indiffvidual (*n* = 4), the sample size for validation experiments was increased to a viable number (*n* = 19 AAA and *n* = 11 control aortae) and showed significant down-regulation of miR-362, -19b-1, -194, -769, -21 and -550 (Figure 2). For the first time, these results were correlated with a basic histological grading of the specimen and revealed an altered expression pattern based on inflammation. This was especially evident for miR-21, possibly explaining the discrepancy with the previously reported up regulation in AAA (Figure 2) [16]. The initial candidate identification is, however, biased by the small sample size, possibly explaining the small overlap between our candidates and previously analyzed miRs (Table 1).

miR-194 and -550 have been reported to be up regulated in lung carcinoma and COPD, hypothetically suggesting a weak relation to tobacco use. Moreover, miR-194 has been extensively studied in carcinogenesis of different types [27]. miR-769 and -362 have never been referred to in the context of AAA. miR-19b-1, solely or clustered, has been linked to apoptosis and angiogenesis and direct targeting of Bcl2 and PTEN has been verified [28]. Interestingly, up-regulation of PTEN was demonstrated to promote AAA expansion [16]. In addition, angiogenesis is seen in the tunica media of AAA tissue, and has gained attention resulting in several anti-angiogenic drugs are now under clinical investigation (Figure S1).

PAAs has never been studied for miR expression. However, comparative analysis with other aneurysm entities such as AAA has the potential to identify core mechanisms in aneurysm development [29]. In PAA, due to limited extension of the dilatation, tissue harvest from aneurysmatic and non-aneurysmatic parts from the same individual is feasible and therefore allows a clear comparison. Albeit similar tissue morphology, expression results were not statistically significant in our analysis, possibly due to the small sample size (*n* = 8) (Figure 2). Expression differences suggest a different nature of VSMCs from muscular and elastic arteries, which, however, might be of interest when investigating further downstream effects of aneurysms, since differences in protein or mRNA levels might be connected. Opposite, inverse expression of miR-550 and -21 in AAA and PAA is of interest and could be localized to VSMCs, the main effector cells in aneurysm disease [16]. To our best knowledge, no commercial human popliteal artery VSMC cell line is available and miR-550 seems to be species-specific to homo sapiens, thus limiting further research.

Target prediction from miR studies is widely accepted. Encouragingly the candidates presented in this study led to similar results as previous suggestions, thus verifying both our results and those of others [21]. Linkage analysis to a coherent mRNA profile was not possible in our study. miR down-regulation, resulting in increased function of the respective gene set, has to be understood in the context of comprehensive pathway analysis. Various genes within different pathways are further targeted by a combination of two or more of the six miRs (data not shown).

Although single gene target prediction is interesting, it is potentially of greater interest to investigate entire signal transduction pathways (Table 1) in aneurysm formation, since the enrichment of miR interaction has the potential for targeted therapy [9,30,31,32]. Cytokine-cytokine interaction, tissue remodeling and cell adhesion molecule pathways have been shown to be enriched in AAA, and some overexpressed adhesion molecules have been assessed as potential biomarkers [33,34]. The less important involvement of pathogen-associated inflammatory pathways is in agreement with the current opinion that AAA is a non-immunogenic driven process [35].

Immunohistochemistry for TGFß and CD31 was done, in order to determine whether the predicted pathways such as canonical TGF signaling, angiogenesis (VEGF mediated) and endothelial processes including adherence/tight junction as well as cell adhesion molecules might have a biological role in the investigated tissues. In this respect, TGFß was less expressed in both aneurysm entities and CD31 showed abundant neoangiogenesis as well as luminal endothelial disruption (Figure S1). However, to conclusively observe differentiation with respective to tissue inflammation and aneurysm entity as well as linkage to differentially expressed miRs is not possible by this means. Further studies are needed to shed light on the specific biological role of these single miRs.

## 3. Experimental Section

Tissue acquisition: Tissue acquisition was in accordance with the declaration of Helsinki, with approval of the local ethic review committee (University of Würzburg, Würzburg, Germany) of this study and with patients’ informed and written consent. 19 AAA, 11 non-aneurysmatic aortae and 8 PAA with their adjacent popliteal artery samples were collected during open surgery. Four of the aortic samples were paired aneurysmatic *vs.* non-aneurysmatic from the same individual for initial miR expression profile. Tissue was immediate rinsed in PBS and divided, where a portion was fixed in formalin (3.5% formaldehyde, Fischar, Saarbrücken, Germany) and the remaining piece was snap frozen in liquid nitrogen.

HE staining and light microscopy: 1 µm sections of paraffin embedded samples were mounted on Superfrost^©^ slides (Menzel, Braunschweig, Germany) and stained for HE. Light microscopy images were made with a Keyence BZ9000 microscope (Keyence, Kyoto, Japan). Image analysis was performed with AZ Analyser II software (Keyence, Kyoto, Japan) distributed by Keyence. AAA samples were then investigated for HISA criteria by two independent researchers and a final decision obtained by consent.

Immunhistochemistry: 1 µm sections of paraffin embedded sections (2 controls, 3 AAA, 3 PAA respectively) were stained for CD31 (M0823, DAKO, Hamburg, Germany, 1:100) and TGFß (ab92486, Abcam, Cambridge, UK, 1:100) respectively on an overnight protocol with Counterstaining done with either nuclear fast red aluminum or Mayer’s hematoxylin (both from Carl Roth, Karlsruhe, Germany). Antigen retrieval was done by 20 min cooking in a ph6 solution. Negative control included PBS incubation instead of primary antibody on the same tissues.

miR extraction: miR extraction of snap frozen tissue specimen was performed using the whole RNA phenol-based mirVana™ miRNA Isolation Kit (Ambion^®^, Life Technologies, Frankfurt, Germany). Approximately 100 mg of tissue were pulverized with pestle and mortar under repeated rinsing with liquid nitrogen over dry ice and then handled according to the manufacturer’s protocol. Sufficient RNA integrity was verified using the Experion automated electrophoresis station (Bio-Rad Laboratories, Munich, Germany).

miR expression profiling: A miRNA expression profile of four patients with AAA and abdominal aortae as control respectively was applied using the TaqMan^®^ Array Human MicroRNA Cards A/B v2.0 (Applied Biosystems^®^, Life Technologies, Frankfurt) along with Megaplex™ RT Primers and TaqMan^®^ MicroRNA Reverse Transcription Kit (Applied Biosystems^®^, Life Technologies, Frankfurt) components according to the manufacturer’s protocol. qRT-PCRs were performed in duplicates with a 7900HT Fast Real-Time™ PCR System (Applied Biosystems^®^, Life Technologies, Frankfurt) with 384-Well Block Module. Small non-coding RNAs U6 and RNU48 served as endogenous controls for normalization [36]. StatMiner^®^ software (Integromics^®^, Madison, USA) was used for data processing and analysis.

miR qRT-PCR: Validation experiments were performed using TaqMan^®^ MicroRNA Assays (Applied Biosystems^®^, Life Technologies) for miRs -769-5p (MIMAT0003886), -550a-5p (MIMAT0004800), -194-5p (MIMAT0000460), -19b-1-5p (MIMAT0004491), -362-3p (MIMAT0004683), -21-3p (MIMAT0004494) and small non-coding RNAs U6 and RNU48 for endogenous control. TaqMan^®^ MicroRNA Reverse Transcription Kit, Universal PCR Master Mix II No UNG and Megaplex™ RT Primers, Human Pool Set v3.0 (Applied Biosystems^®^, Life Technologies, Frankfurt) were applied accordingly. The cDNA program was run on a thermal cycler Primus 96 (Peqlab Biotechnologie, Erlangen, Germany) and the qRT-PCR was run in biological duplets on a CFX96 real-time PCR system with CFX Manager Software 2.0 (Bio-Rad Laboratories, Hercules, CA, USA).

miR in-situ-hybridization: miR-550 and miR-21 hybridization was performed on formalin-fixed samples of AAA and PAA as well as control tissues with miRCURY LNA™ microRNA Detection Probes for *In Situ* Hybridization^®^ (Exiqon, Vedbaek, Holland) according to the manufacturer’s protocol with a final concentration of the detection probe of 25 and 20 nM respectively.

DIANA miRPath analysis: DIANA lab (http://diana.cslab.ece.ntua.gr) developed algorithms and tools for interpreting genomic data with special interest in miRNA [37]. The DIANA miRPath web server utilizes miRNA target prediction with high accuracy based on DIANA-microT-CDS and verified targets from miRTarBase v6.0 (http://mirtarbase.mbc.nctu.edu.tw) along with clustering of miRNAs and pathways based on KEGG database interaction. A specific analysis for the identified miRs and previously described miRs was performed. The resulting predicted interactions were organized in the context of KEGG pathway.

Statistics: Microsoft Excel was used to perform a Welch’s *t*-test for unpaired samples of small numbers (AAA) and paired samples (PAA). A level of ≤0.05 was considered significant. The fold change of miRNA expression in the various samples was calculated by the ΔΔ*C*_t_ method with relative quantities as log_10_RQ = log_10_2^−Δ(Δ*C*t)^ [38]. All graphs were made with Microsoft Excel.

## 4. Conclusions

While the role of miRs for future diagnostic and treatment purposes is still vague, the number of proposed candidates from tissue signature studies continues to increease and application possibilities continue to further diversify, taking into account heterogeneous tissue morphology.

Studying miR expression in two distinct entities, AAA and PAA, and combining this to form a histological assessment of the tissue involved, helps us understand the core mechanisms of aneurysm formation, both in concordantly and inversely altered pathways. Enrichment analysis of miR-362, -19b-1, -194, -769, -21 and -550 indicated the involvement of signal transduction, cell–cell interaction and cell degradation as general mechanisms, but further research is needed to further decipher processes in relation to aneurysm pathology.

## Supplementary Materials

Supplementary materials can be found at http://www.mdpi.com/1422-0067/17/1/81/s1.
