# Supplementary material for: Aneurysm miRNA Signature Differs, Depending on Disease Localization and Morphology
Source: Int J Mol Sci. 2016 Jan 12;17(1):81. doi: 10.3390/ijms17010081 (PMC4730325; doi:10.3390/ijms17010081)
Supplement: Supplementary file 1 [file ijms-17-00081-s001.pdf]

Supplementary Materials: Aneurysm miRNA Signature Differs, Depending on Disease Localization and Morphology

Albert Busch, Martin Busch, Claus-Jürgen Scholz, Richard Kellersmann, Christoph Otto, Ekaterina Chernogubova, Lars Maegdefessel, Alma Zernecke and Udo Lorenz

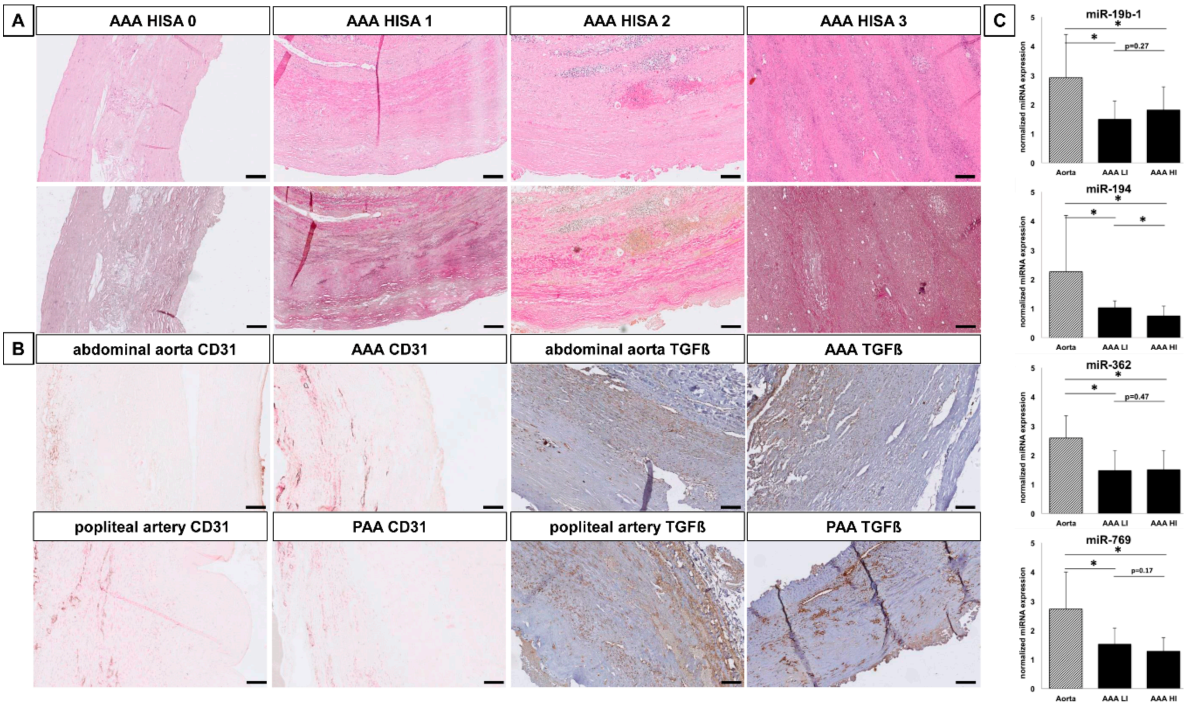

Figure S1. Histologic inflammation scale of aneurysm (HISA) scheme, TGFß and angiogenesis immunohistochemistry and miR expression inabdominal aortic aneurysm (AAA): Detailed histologic analysis allows categorization of human AAA samples to the HISA scheme based on inflammation and fibrosis with a range from no inflammation (HISA0) to severe chronic inflammation (HISA3) (**A**). HISA0 and HISA1 are considered low inflammatory AAA and approx. 60% of specimen filled these criteria vs. high inflammatory HISA2 and HISA3. The latter show a markedly thickened intima/media layer with an average of approx. 3 mm double as thick on histologic sections than low inflammatory specimen; (**B**) AAA and popliteal aneurysm (PAA) show marked angiogenesis by CD31 positive microvessels in the adventitia and media/neointima. Endothelial disruption in aneurysm samples is also seen, as the luminal endothelium (pointing right) is not CD31 positive. Staining for TGFß in vascular smooth muscle cells (VSMC) of control and aneurysmal arteries suggests less protein in aneurysm tissues. Patient data from PAA/popliteal artery samples are similar to AAA data; (**C**) Additional inflammatory dependent expressions results for miRs-19b-1, -194, -362, -769 shows miR-194 expression in AAA tissue to be dependent on the grade of inflammation. (* = $p < 0.05$; magnification 10×; scale bar 50 µm).

Table S1. Patients' characteristics.

Patient Characteristic	AAA *n* = 42	HISA 0 *n* = 3 (7%)	HISA 1 *n* = 17 (39%)	HISA 2 *n* = 15 (35%)	HISA 3 *n* = 7 (14%)	Control Aorta *n* = 13	PAA/Control Popliteal Artery *n* = 15 PAA from *n* = 12 Individuals
Age (a)	70 ± 8.2	70 ± 7.9	72 ± 17	70 ± 7.4	66 ± 12.2	68 ± 8.8	68.5 ± 8.7
Sex (f/m)	4:38	0:3	3:14	1:15	0:7	3:10	0/12
Vessel diameter (mm)	66 ± 18	51 ± 2	69 ± 17	65 ± 21	73 ± 11	<30	29 ± 4 (norm <10)
Bilateral PAA	-	-	-	-	-	-	66.7%
Rupture	14.3%	0	2/17	2/15	2/3	-	-
Leukocyte count (×10³/μL)	10.1 ± 2.3	<9	9.7	10.67	10.1	<9	<9 (norm <9)
CRP (mg/dL)	1.06 ± 3.15	<0.4	<0.4	0.81 ± 1.07	3.67 ± 7.3	<0.4	-
HISA acute inflammation	13.9%	0	0	26.7%	33.3%	-	-
Other aneurysm	-	-	-	-	-	-	33.3%
Other comorbidities							
Hypertensive disease	58.0%	33.3%	70.6%	66.7%	57.1%	72.3%	66.7%
NIDDM	21.1%	0	41.2%	13.3%	0	27.3%	25%
CAD	21.1%	33.3%	17.6%	20%	28.6%	-	16.7%
Smoking history	94.7%	100%	47.1%	46.7%	71.4%	72%	83%
Renal disease (KDIGO > 1)	26.3%	0	29.4%	33.3%	28.6%	9.1%	-
Stroke history	9.5%	0	0	20%	14.3%	-	33.3%
COPD	11.9%	0	5.9%	26.7%	0	-	16.7%

Patients' characteristics on aneurysm and control tissue samples: Table depicts detailed patients' characteristics and co-morbidities (AAA = abdominal aortic aneurysm; HISA = histologic inflammation scale of aneurysm; PAA = popliteal artery aneurysm; n = number; a = anno (years); f = female; m = male; norm = normal value; NIDDM = non-insulin dependent diabetes mellitus; CAD = coronary artery disease; KDIGO = kidney disease improving global outcomes; COPD = chronic obstructive pulmonary disease).
